# Overexpression of Chicken IRF7 Increased Viral Replication and Programmed Cell Death to the Avian Influenza Virus Infection Through TGF-Beta/FoxO Signaling Axis in DF-1

**DOI:** 10.3389/fgene.2018.00415

**Published:** 2018-09-25

**Authors:** Tae Hyun Kim, Huaijun Zhou

**Affiliations:** ^1^Department of Animal Science, University of California, Davis, Davis, CA, United States; ^2^Integrative Genetics and Genomics Graduate Group, University of California, Davis, Davis, CA, United States

**Keywords:** AIV, avian influenza, chicken, DF-1 cell line, IRF7, overexpression, RNA-seq

## Abstract

During mammalian viral infections, interferon regulatory factor 7 (IRF7) partners with IRF3 to regulate the type I interferon response. In chickens, however, it is still unclear how IRF7 functions in the host innate immune response, especially given that IRF3 is absent. To further elucidate the functional role of chicken IRF7 during avian influenza virus (AIV) infection, we generated inducible IRF7 overexpression DF-1 cell lines and performed *in vitro* infection using low pathogenic AIVs (LPAIVs). Overexpression of IRF7 resulted in higher viral replication of H6N2 and H10N7 LPAIVs compared to empty vector control cells regardless of IRF7 expression level. In addition, a high rate of induced cell death was observed due to elevated level of IRF7 upon viral infection. RNA-seq and subsequent transcriptome analysis of IRF7 overexpression and control cells discovered candidate genes possibly controlled by chicken IRF7. Functional annotation revealed potential pathways modulated by IRF7 such as TGF-beta signaling pathway, FoxO signaling pathway and cell structural integrity related pathways. Next, we analyzed the host response alteration due to the IRF7 overexpression and additionally discovered the possible connection of chicken IRF7 and JAK-STAT signaling pathway. These findings suggest that chicken IRF7 could modulate a wide range of cellular processes in the host innate immune response thus meticulous control of IRF7 expression is crucial to the host in response to AIV infection.

## Introduction

Avian influenza virus (AIV) is one of the major pathogens that significantly impacts the poultry industry worldwide (Olsen et al., 2006; Swayne, 2012). For example, recent high pathogenic avian influenza (HPAI) outbreaks between late-2014 to mid-2015 in the United States resulted in the death of more than 50 million birds, an estimated 12% of the layer chickens and eight percent of the meat turkeys raised in the United States that year (Jhung and Nelson, 2015; Ramos et al., 2017). Declined production and HPAI-related trade restrictions further contributed to the significant economic loss to the industry (Ramos et al., 2017). Current strategies for controlling AIV primarily rely on passive measures such as quarantine and slaughter, partially due to our limited understanding of the chicken antiviral response compared to mammals (Goossens et al., 2013). A better understanding of the host antiviral response in chickens could provide critical information to develop improved prevention strategies as well as novel therapeutics against AIV (Downing et al., 2009; Magor et al., 2013).

Interferons (IFNs) are known to trigger host innate immune responses against viral infection by activating signal transduction pathways (Der et al., 1998; de Weerd et al., 2007), and currently more than 3,800 IFN regulated genes have been reported according to the Interferome database (Rusinova et al., 2013). IRF7 is well known as the master transcription factor that interacts with IRF3 to initiate the type I IFN response in mammals (Honda et al., 2005; Honda and Taniguchi, 2006). Even though chickens can induce type I IFNs robustly in response to viral infection (Der et al., 1998), avian species lack IRF3, and the precise molecular and cellular mechanisms of IFN regulation remain to be elucidated (Huang et al., 2010; Santhakumar et al., 2017). Based on this, we hypothesized that the chicken IRF7 may have a conserved function in regulating antiviral response in chickens, yet the signaling cascade and mechanism of action could be species-specific.

In our recent analysis of stable overexpression and knockdown of IRF7 in chicken DF-1 cell lines followed by mimicking viral infection with dsRNA analog poly(I:C), we demonstrated that the primary function of IRF7 as type I IFN regulator may be conserved (Kim and Zhou, 2015). Constitutive overexpression of IRF7 resulted in upregulation of *IFNB* upon poly(I:C) induction whereas IRF7 knockdown caused downregulation of *IFNA* (Kim and Zhou, 2015). Further transcriptome analysis revealed more than 60 novel candidate genes that are potentially regulated by IRF7, suggesting a distinct function of chicken IRF7 (Kim and Zhou, 2015). Another study demonstrated that the knockdown of IRF7 by siRNA limited *IFNA*, *IFNB*, and *STAT1* mRNA expression and increased Newcastle disease virus replication in chicken embryonic fibroblasts (CEFs), suggesting the functional role of IRF7 as a type I IFN regulator (Wang Y. et al., 2014).

To further elucidate the functional role of chicken IRF7 in the context of AIV infection, we took advantage of the inducible expression system to control the expression level of IRF7 in DF-1 cells and infected the established cell lines with two low pathogenic AIV (LPAIV) strains. Correlation between the IRF7 expression level and the AIV replication phenotype was investigated with different levels of IRF7 induction. In addition, we analyzed the transcriptome of IRF7 overexpression and control cells by RNA-seq after LPAIV or mock infection to examine candidate genes and pathways that are potentially modulated by IRF7 upon AIV infection.

## Materials and Methods

### Expression Plasmid Construction

**FIGURE 1:**
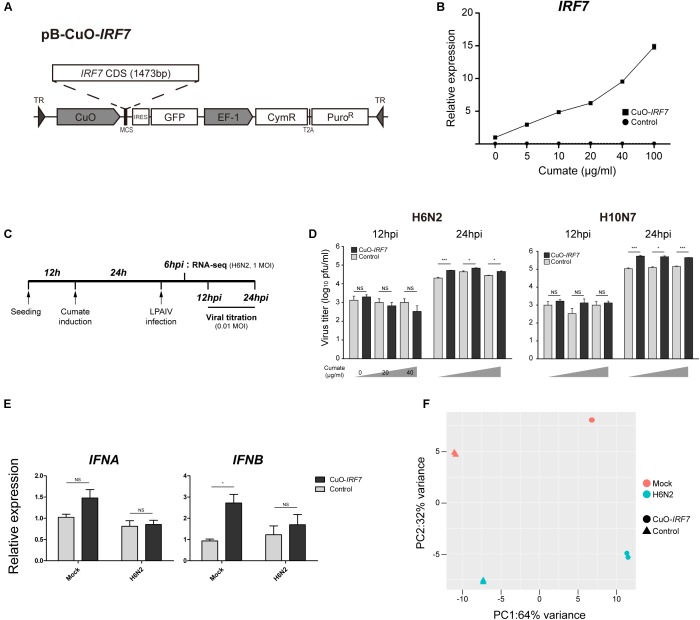
Characterization of IRF7 inducible overexpression in DF-1 cell line **(A)** Schematic diagram of IRF7 inducible overexpression plasmid (CuO-*IRF7*). Empty vector plasmid was used to generate proper control (Control). **(B)** Titratable IRF7 expression induction. IRF7 expression induction was measured by qRT-PCR at 24 h post-induction of cumate. All data are shown as mean ± SEM from three biological replicates. **(C)** Experimental timeline of *in vitro* low pathogenic avian influenza virus (LPAIV) infection. Cuo-*IRF7* and Control cell lines were induced by various level of cumate (0, 20, 40 μg/ml) at 12 h after passaging for 24 h. Induced cell lines were infected with H6N2 or H10N7 [0.01 MOI (multiplicity of infection)] and progeny viral titer was measured by plaque assay at 12 and 24 hpi. For RNA-seq, cell lines were infected by either mock or H6N2 with an MOI of 1 and cells were harvested at 6 hpi. **(D)** Progeny viral production in the media was measured by plaque assay. All data are shown as mean ± SEM (*N* = 3, ^∗^*p* < 0.05, ^∗∗∗^*p* < 0.001, NS: not significant; Two tailed *T*-test). **(E)** Expression of type I IFNs in the CuO-*IRF7* and control cell lines in mock and upon H6N2 infection (6 hpi, 1 MOI). Relative expression levels of IFNA and IFNB were measured by qRT-PCR. All data are shown as mean ± SEM from three biological replicates (^∗^*p* < 0.05, NS, not significant; Two tailed *T*-test). **(F)** Principal component analysis (PCA) of cDNA libraries. Replicates from each experimental group is separated by IRF7 expression level and H6N2 infection.

Chicken *IRF7* coding sequence (CDS, KP_096419) was cloned into the piggyBac(pB) cumate expression inducible plasmid (System Biosciences, Mountain View, CA, United States) which controls the expression level by cumate gene switch (pB-CuO-*IRF7*). The inducible vector co-expresses the repressor, CymR and puromycin resistance gene driven by consecutive EF1α promoter (**Figure 1A**). Consistently expressed CymR binds to the CuO promoter to repress gene expression and addition of cumate changes the conformation of CymR which then turns on the gene switch by removing the repressor from the promoter.

### Inducible IRF7 Overexpression Cell Line Establishment

Immortalized chicken embryonic fibroblast DF-1 cells (ATCC, Manassas, VA, United States) were cultured in Dulbecco’s modified Eagle’s medium (Thermo Fisher Scientific, Waltham, MA, United States) supplemented with 10% fetal bovine serum (Thermo Fisher Scientific, Waltham, MA, United States), 1x Antibiotic-antimycotic (Thermo Fisher Scientific, Waltham, MA, United States), and incubated at 37°C in a humidified atmosphere containing 5% CO_2_. Empty vector (Control) or chicken IRF7 inducible expression vector (pB-CuO-*IRF7*) was co-transfected with pB transposase plasmid into DF-1 cell lines using the Lipofectamine 3000 reagent (Thermo Fisher Scientific, Waltham, MA, United States) according to the manufacturer’s protocol for efficient integration. Puromycin (3 μg/ml) was added to the culture media 48 h after transfection and stably integrated cell lines were selected for 2 weeks. To induce *IRF7* expression, cumate (4-Isopropylbenzoic acid, Sigma-Aldrich, St. Louis, MO, United States) was added to the culture media at 12 h after seeding for 24 h followed by subsequent *in vitro* experiments.

### Quantitative Reverse Transcriptase PCR

Total RNA was isolated from approximately 1 million cells using Direct-zol RNA MiniPrep Kit (Zymo Research, Irvine, CA, United States) and complement DNA (cDNA) was synthesized from total RNA (500 ng) using Verso cDNA Synthesis Kit (Thermo Fisher Scientific, Waltham, MA, United States). Quantitative reverse transcriptase PCR (qRT-PCR) was performed using the Applied Biosystems 7500 Fast Real-Time PCR System (Life Technologies, Grand Island, NY, United States) with SYBR Select Master Mix (Life Technologies, Grand Island, NY, United States). *IRF7, IFNA, IFNB* expression was normalized to the chicken glyceraldehyde 3-phosphaste dehydrogenase (*GAPDH*) gene using the ΔΔ C_T_ method (Livak and Schmittgen, 2001; Kim and Zhou, 2015).

### Virus and *in vitro* AIV Infection

A/Chicken/California/2000 (H6N2) and A/Chicken/California/1999 (H10N7) low pathogenic avian influenza virus (LPAIV) strains were kindly provided by Dr. Rodrigo Gallardo (University of California, Davis, CA, United States) and Dr. Peter Woolcock [University of California, Davis, California Animal Health and Food Safety (CAHFS)], respectively. Each LPAIV was propagated in Madin-Darby Canine Kidney (MDCK) cells as described in Eisfeld et al. (2014). All *in vitro* AIV infections were performed using CellBIND 12 well tissue culture plate (Corning, NY, United States) with 1 × 10^6^ cells per well at the seeding. For viral replication kinetics phenotype, established DF-1 cell lines were induced and infected with either H6N2 or H10N7 at multiplicity of infection (MOI) of 0.01 with 0.05 μg/ml TPCK-trypsin in DMEM. Culture supernatants were collected at 0, 12, and 24 h post-infection (hpi) and the viral titer of each sample was measured by the plaque assay using MDCK cells (Huprikar and Rabinowitz, 1980). For the transcriptome profiling study, non-induced cell lines were infected with either mock or H6N2 at an MOI of 1 with 0.05 μg/ml TPCK-trypsin in DMEM. Trizol reagent (Thermo Fisher Scientific, Waltham, MA, United States) was directly added to the cell monolayer after washed twice with PBS to extract the total RNA at 6 hpi which the IRF7 expression level starts to peak upon AIV infection (Kim and Zhou, 2015).

### RNA Sequencing and Data Analysis

A total of eight cDNA libraries were prepared from two biological replicates of each group (mock or H6N2 infected Control or CuO-*IRF7*). RNA sequencing libraries were prepared from poly-adenylated RNA and sequenced with Illumina HiSeq4000 which generated over 20 million 150 bp paired-end reads per sample. The read files from RNA-seq analysis have been deposited in NCBI’s Gene Expression Omnibus with GEO Series accession number GSE115131. We checked the quality of each library by fastQC (version 0.11.6) and trimmed the adaptor sequence by TrimGalore (version 0.4.5). We aligned the trimmed fastq files to the galGal5 chicken genome using STAR aligner (version 2.6.0) with NCBI annotation release 103 (Dobin et al., 2013). Unmapped reads were aligned against H6N2 (A/chicken/CA/6643/2001) genome (Webby et al., 2002). Raw read counts were extracted by HTSeq (version 0.9.0) from each aligned bam files and used to identify differentially expressed genes (DEGs) (Anders et al., 2015). Both DESeq2 and EdgeR R packages were used to identify DEGs and the DEG sets from the both packages were combined (false discovery rate (FDR) < 0.1% in any one of the packages) (Robinson et al., 2010; McCarthy et al., 2012; Love et al., 2014). The combined DEG lists were further filtered by removing the low expression genes if both samples of given comparison had fragments per kilobase per million reads (FPKM) value less than 1. Functional annotations for significantly DEGs were performed using DAVID 6.8 (Dennis et al., 2003; Huang da et al., 2009). The enriched gene ontology (GO) terms on biological processes and the pathways obtained from DAVID functional analysis were filtered for significance by gene count ≥ 5 and *p*-value < 0.05.

### Hoechst 33342/Propidium Iodide Staining

Each control or CuO-*IRF7* cells were seeded at 1 × 10^5^ cells/well to attachment factor protein (Thermo Fisher Scientific, Waltham, MA, United States) treated μ-Plate 96 Well glass bottom plate (ibidi GmbH, Germany) before 24 h of infection and infected 1MOI with three independent replicates. At 6 and 12 hpi, each well was washed twice with PBS and stained with 1 μg/ml each of Hoechst 33342 (Thermo Fisher Scientific, Waltham, MA, United States) and propidium iodide (Thermo Fisher Scientific, Waltham, MA, United States) in PBS at room temperature for 10 min. Two images were taken from each replicate by using 20× objective lens and captured by Nikon NIS 3.0 software. A total number of cells (blue) and dead cells (red) were counted using ImageJ (Wayne Rasband, National Institutes of Health, United States) software from all images.

## Results

### Inducible Over-Expression of IRF7 in DF-1 Cell Line

To precisely control the expression level of IRF7 *in vitro*, stable IRF7 inducible overexpression (CuO-*IRF7*) DF-1 cells were established using a cumate inducible vector (**Figure 1A**). Empty vector control cells (Control) which have identical expression cassettes without IRF7 CDS were also generated to exclude potential random gene disruption noise due to vector integration. We induced established cell lines with various cumate concentrations from 0 to 100 μg/ml for 24 h to titer the expression level of IRF7 by qRT-PCR (**Figure 1B**). There was approximately 10-fold higher IRF7 expression in CuO-*IRF7* cells compared to the control cells without induction (**Supplementary Figure S1**). Titratable *IRF7* expression was observed in CuO-*IRF7* cells as cumate concentration increases (up to 15-fold upregulation vs. non-induced cells) and the induction levels of *IRF7* were highly reproducible. In contrast, IRF7 expression level was not affected by cumate induction in control cells.

### LPAIV Virus Replication in the Induced Cell Lines

Two LPAI virus strains H6N2 and H10N7 at an MOI of 0.01 were used to infect the cumate induced cell lines, and the correlation between IRF7 expression level and the AIV replication was analyzed. Both CuO-*IRF7* and control cell lines were induced with 0, 20, or 40 μg/ml of cumate for 24 h which correspond to approximately 10, 50, and 100-fold overexpression of *IRF7* in CuO-*IRF7* cells compared to the control cells at the time of infection. Then, AIV replication dynamics were analyzed at 12 and 24 h post-infection (hpi) by MDCK based plaque assay (**Figure 1C**). At 12 hpi, there was no significant difference in viral replication observed between *IRF7* overexpression and control cells for both LPAI virus strains at any level of induction. At 24 hpi, we observed significantly increased viral replication in IRF7 overexpressed cell lines compared to the control cell lines from the both virus strains with a range of 1.6-fold (H6N2, 20 μg/ml) up to fivefold (H10N7, 0 μg/ml) (**Figure 1D**). However, we did not observe any significant correlation between the induced IRF7 expression level and the viral titer in the overexpression cells.

### Type I IFN Regulation by IRF7

We additionally measured the expression levels of type I IFNs in mock or H6N2 infected (1 MOI) control and overexpression cells to examine the type I IFN regulator role of IRF7 (**Figure 1E**). In mock infected cell lines overexpression of IRF7 resulted almost threefold upregulation of IFNB whereas IFNA level did not show significant upregulation. There was no significant difference in expression of *IFNA* and *IFNB* between the Control and Cuo-*IRF7* cells upon infection despite upregulation of IRF7 upon infection in each cell line (Control: 1.44-fold, FDR = 0.039; Cuo-*IRF7*: 1.31-fold, FDR = 0.098, DESeq2).

### Transcriptome Analysis

To identify the candidate genes and signaling pathways that IRF7 may regulate during AIV infection, we performed RNA-seq analysis on four different experimental groups of cells. There was no significant difference in viral replication among different IRF7 overexpression levels, thus non-induced DF-1 cells with either mock or H6N2 infection treatments were used and harvested at 6 hpi (**Figure 1C**). Each condition had 2 independent biological replicates and principal component analysis showed that the replicates in each condition grouped together by either IRF7 expression level or H6N2 infection condition (**Figure 1F**). The alignment rate against galGal5 reference genome was on average of 84.98% from mock infection libraries and decreased to 71.57% with H6N2 infection as approximately 15% of the total reads were mapped to the viral genome in H6N2 infected cell lines.

### Differential Expression Analysis of IRF7 Overexpression

**FIGURE 2:**
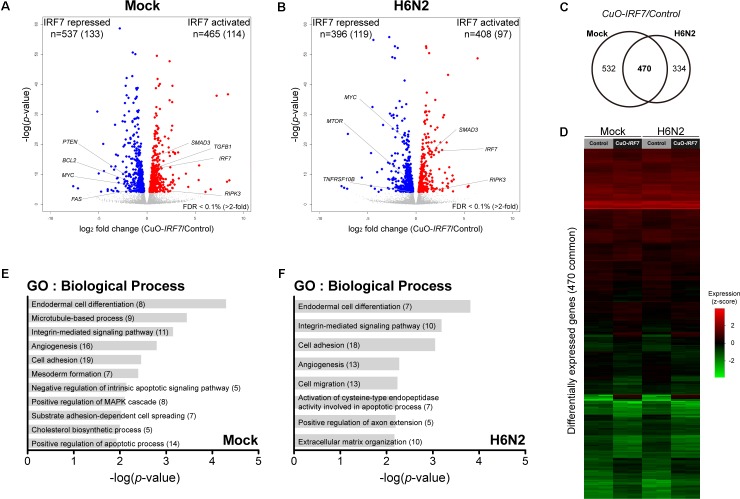
IRF7 overexpression altered transcriptome regulation in DF-1 cells. Volcano plot of transcriptomic difference between Control and CuO-*IRF7* cells in **(A)** mock infection and **(B)** H6N2 infection conditions; colored dots correspond to significant DEGs [False discovery rate (FDR) < 0.1%]. Number of differentially expressed genes (DEGs) are shown at the top of each plot and number of genes that have more than twofold change is in the parenthesis. **(C)** Venn diagram of DEGs between mock and H6N2 infected condition. **(D)** Heatmap showing relative gene expression [log2FPKM (fragments per kilobase million)] of 470 common DEGs reflected in **(C)** with all samples. Each row represents a DEG and each column represents a sample. Gene ontology (GO) analysis by DAVID 6.8 using DEGs from **(E)** mock infection and **(F)** H6N2 infection condition. Number of genes enriched in each biological process is in parentheses.

**FIGURE 3:**
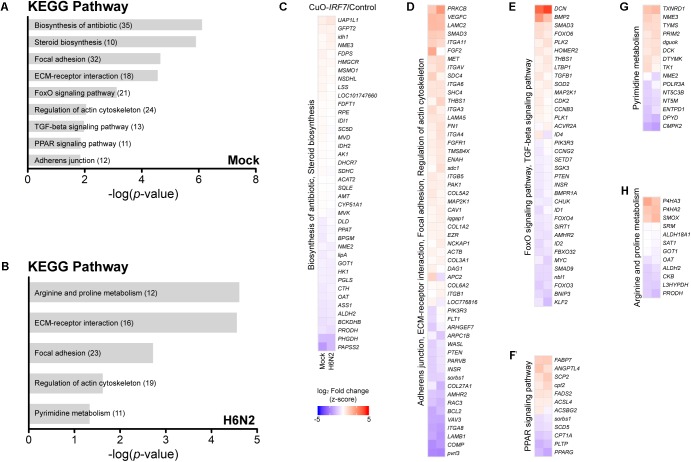
Pathway analysis of DEGs and their expression patterns. Pathway analysis enrichment using DEGs between Control and CuO-*IRF7* cells in **(A)** mock infection and **(B)** H6N2 infection conditions. Number of genes in each pathway is in parentheses. **(C–H)** Heatmaps showing the expression fold change of DEGs in significantly enriched pathways in mock infection (left column) and H6N2 infection (right column). Pathways were combined based on the common genes across the pathways. Lists of DEGs from both conditions in each pathway were combined to generate each heatmap.

First, by directly contrasting the transcriptomes between the control and *IRF7* overexpression cell lines, we were able to identify the DEGs that were potentially regulated by IRF7 either at basal condition or upon H6N2 infection. There were 1,002 DEGs (465 up-, 537 down- regulated) in mock condition and 804 DEGs (408 up-, and 396 down-regulated) in H6N2 infection (**Figures 2A,B** and **Supplementary Table S1**). Comparison of the two DEG lists showed 470 genes were overlapped between mock and infection conditions (**Figure 2C**). The gene expression heatmap showed distinct gene expression pattern difference between control and CuO-*IRF7* cells among the common 470 DEGs (**Figure 2D**). Gene ontology (GO) analysis from all DEGs revealed enriched functions involved in cell structural integrity or cellular assembly such as cell adhesion, extracellular matrix (ECM) organization, integrin signaling mediated processes, and apoptosis (**Figures 2E,F**). Furthermore, we performed pathway analysis with the same gene sets to examine which pathways could be potentially altered as a result of IRF7 overexpression. Antibiotic and steroid biosynthesis pathways were significantly enriched in the mock condition while arginine, proline, and pyrimidine metabolisms were significantly enriched upon infection (**Figures 3A,B**). In addition, cell structure and ECM related pathways were enriched in both conditions (**Figures 3A,B**). Pathway analysis also revealed that FoxO signaling, TGF-beta signaling and PPAR signaling pathways, which are related to the immuno-regulation, could be possibly regulated by IRF7 (**Figure 3A**). DEGs in FoxO signaling and TGF-beta signaling pathways are presented in **Supplementary Figure S2**. Heatmaps with fold changes of all individual DEGs from significantly enriched pathways are presented in **Figures 3C–H**). Most of the integrin (ITG), laminin (LAM), and collagen (COL) DEGs were up-regulated in both conditions except for *ITGA9*, *LAMB1*, and *COL6A2*, which were down-regulated DEGs (**Figure 3D**). *TGFB1* and *SMAD3* were up-regulated whereas *PTEN* and *MYC* were down-regulated in the TGF-beta signaling pathway in both conditions (**Figure 3E**).

### IRF7 Overexpression Increased Cell Death Upon AIV Infection

**FIGURE 4:**
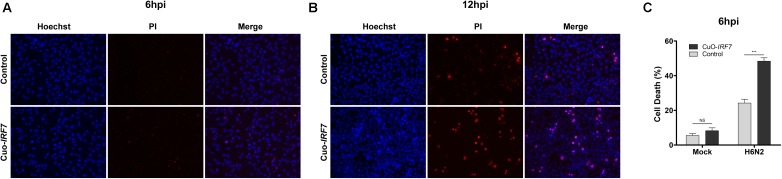
IRF7 overexpression resulted more cell death. Representative images of Hoechst 33342 and Propidium Iodide (PI) double nuclear staining of control and Cuo-*IRF7* cell lines at **(A)** 6 h (*N* = 6) and **(B)** 12 h (*N* = 3) post-infection with H6N2. Images were visualized by 20x subjective lens. **(C)** The percentage of dead cells (PI positive; red) compared with total cells (Hoechst positive; blue) for Control and CuO-*IRF7* cells with mock and H6N2 condition at 6 hpi. All data are shown as mean ± SEM from six replicates. (^∗∗∗^*p* < 0.001, Two tailed *T*-test).

Based on the enriched GO terms and signaling pathways, we further investigated the effect of IRF7 overexpression on cell viability using Hoechst 33342/PI nucleic acid staining fluorescence microscopy. While Hoechst dyes can penetrate the living cells, propidium iodide only stains dead cells. Representative images of Hoechst 33342/PI staining are shown in **Figure 4A** (6 hpi) and **Figure 4B** (12 hpi). Approximately twice as many CuO-*IRF7* cells (48.4 ± 5.3%) died upon H6N2 infection at 6dpi as control cells (24.2 ± 4.7%) at 6 hpi (**Figure 4C**).

### Role of IRF7 in the Host Response Against H6N2 Infection

**FIGURE 5:**
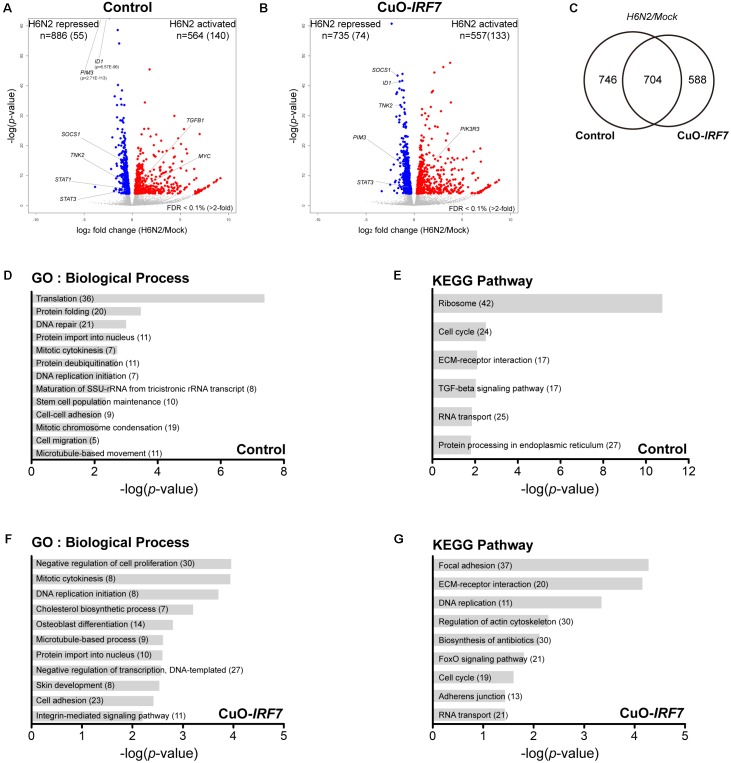
Transcriptome analysis of host response against H6N2 infection. Volcano plot of transcriptomic differences between mock and H6N2 infection conditions in **(A)** Control cells and **(B)** CuO-*IRF7* cells; colored dots correspond to significant DEGs (FDR < 0.1%). Number of differentially expressed genes (DEGs) are shown at the top of the plot and number of genes have more than twofold change is in the parenthesis. **(C)** Venn diagram of DEGs between Control and CuO-*IRF7* cells upon H6N2 infection. Functional analysis Gene ontology and pathways analysis was performed using DEGs from Control; **(D,E),** respectively, and CuO-*IRF7*; **(F,G)**, respectively. Number of genes enriched in each biological process and pathway is in parentheses.

Next, we compared the host responses against H6N2 infection between the cell lines (Control/Mock vs. Control/H6N2 and Cuo-*IRF7*/Mock vs. Cuo-*IRF7*/ H6N2) to investigate which genes and pathways were potentially modulated by IRF7 upon infection (**Supplementary Table S2**). We identified 564 activated genes and 886 repressed genes in control cells upon H6N2 infection (**Figure 5A**) and a similar number of DEGs were identified from overexpression cells (**Figure 5B**; 557 activated genes, 735 repressed genes). There were 704 genes that were common between the two contrasts, and 746 and 588 genes were unique DEGs to the control and Cuo-*IRF7* contrast, respectively (**Figure 5C**). GO and pathway analysis using DEGs from the control contrast represented the molecular signature of host response against AIV infection (**Figures 5D,E**) while the Cuo-*IRF7* contrast could identify unique genes and signaling pathways associated with AIV infection that were potentially regulated by IRF7 (**Figures 5F,G**). Yet, despite the difference in functional annotation observed between the two contrasts, the individual genes did not show dramatic fold change differences between them (**Supplementary Figure S3**).

**FIGURE 6:**
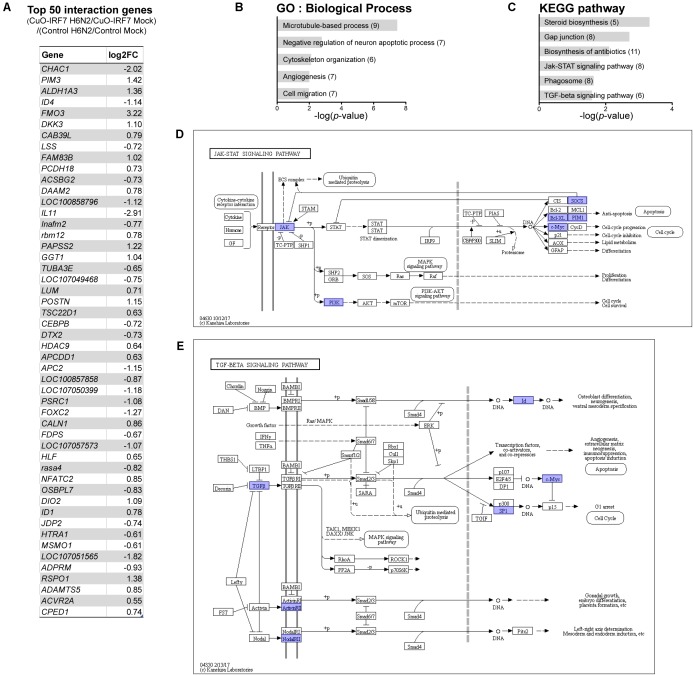
Differentially regulated genes by IRF7 overexpression upon H6N2 infection. **(A)** Top 50 interaction effect DEGs by significance. Functional analysis of interaction effect DEGs was performed for enriched GO **(B)**, and pathway **(C)**. Each interaction effect DEGs are indicated in **(D)** JAK-STAT signaling pathway and **(E)** TGF-beta signaling pathway.

Then, we further analyzed the interaction of IRF7 overexpression by H6N2 infection to identify the genes that were differentially regulated during the infection by IRF7 (**Supplementary Table S3**). The top 50 interaction genes from a total of 350 DEGs (FDR < 5%) are listed in **Figure 6A**. GO analysis (**Figure 6B**) demonstrated that IRF7 overexpression could affect genes in cellular assembly, organization and structural functions as well as apoptosis. Pathway analysis (**Figure 6C**) also showed consistency of the candidate pathways enriched from previous analyses shown in **Figures 3A,B**, **5F,G**. Of particular note, Janus kinase/signal transducers and activators of the transcription (JAK-STAT) signaling pathway were significantly represented in this analysis which was not enriched in any of the above direct contrast functional annotations (**Figure 6D**). In addition, the TGF-beta signaling pathway was also significantly enriched (**Figure 6E**).

## Discussion

Here, we employed a functional genomics approach to investigate the functional role of chicken IRF7 in the host innate immune response to AIV infection by generating an *in vitro* inducible overexpression model followed by whole transcriptome sequencing. Overexpression of IRF7 resulted in higher viral replication as well as greater cell death in our *in vitro* model. The transcriptome analysis suggested that chicken IRF7 might be involved in modulating a wide range of cellular processes including programmed cell death via the TGF-beta, FoxO, and JAK-STAT signaling pathways.

In this study, a cumate inducible system was applied to fine control the overexpression of IRF7 (a 10-fold change by qRT-PCR, and a twofold change by RNA-seq) and its range may better reflect actual physiological expression of IRF7 (**Figure 1B**) compared to the constitutive overexpression system we developed in our previous study that resulted in almost 200-fold overexpression (Kim and Zhou, 2015). Even with this substantially lower IRF7 overexpression level compared to the previous study, we were able to observe the significant *IFNB* upregulation (**Figure 1E**). This further supports a regulatory role of IRF7 on type I IFNs in chickens (Kim and Zhou, 2015). Furthermore, the relatively lower level of upregulation or repression patterns of IFNs upon H6N2 infection despite upregulation of IRF7 could suggest that manipulation of the host immune system by AIV affects the link between the IRF7 and type I IFNs (Alcami and Koszinowski, 2000).

Interestingly, IRF7 overexpression resulted in significantly higher viral titer in the overexpression cells than control cells regardless of IRF7 induction level (**Figure 1D**). However, it is yet to be determined whether the higher progeny viral titer resulting from IRF7 overexpression is detrimental or beneficial to the host as we also observed increased levels of induced cell death (**Figure 4**). Programmed cell death such as apoptosis and necroptosis are host defense strategies known to limit viral infection by eliminating the environment for viruses to replicate (Upton and Chan, 2014; Orzalli and Kagan, 2017), while influenza viruses adapted to inhibit the apoptosis process to benefit their survival through modulating the host response (Zhirnov et al., 2002; Ehrhardt et al., 2007). On the other hand, influenza virus can induce immense host cell death for effective replication and transmission that results in morbidity, pathogenesis and virulence (Brydon et al., 2005; Ludwig et al., 2006). Further *in vivo* investigation could test the hypothesis that IRF7 overexpression was beneficial to the host by promoting the programmed cell death to limit the virus.

Pathway analysis suggested both the TGF-beta and FoxO signaling pathways as potential mechanisms that could be modulated by chicken IRF7 in the host response to AIV. The TGF-beta signaling pathway has diverse functions in cells and tissues, including cell-cycle control, differentiation, extracellular matrix formation, and apoptotic activation (Massague and Chen, 2000; Schuster and Krieglstein, 2002). The FoxO signaling pathway also plays important roles in metabolism, stress resistance, cellular proliferation, and apoptosis by transcription factor family Forkhead box (FOX) proteins. TGF-beta and FoxO signaling pathways are often considered together as their mechanisms of action are closely associated (Naka et al., 2010; Zhang et al., 2011; Wang Z. et al., 2014). TGF-beta induces apoptosis by SMAD-dependent manner (Jang et al., 2002; Schuster and Krieglstein, 2002), and we observed upregulation of *TGFB1* and *SMAD3* and downregulation of anti-apoptotic gene *BCL2* by IRF7 overexpression. This reflects the increased apoptosis and implies IRF7 regulation of the process (**Figures 2A,B**, **3E** and **Supplementary Figure S2**). Qing et al. (2004) suggested the regulation of IRF7 function by TGF-beta/Smad3 signaling as a possible mechanism of the host type I IFN response in the mouse embryonic fibroblasts, yet our results suggest a possible regulation of TGF-beta/Smad3 signaling by chicken IRF7.

FOXO3 was shown as a negative regulator of IRF7 gene transcription in the mouse macrophages in which FOXO3 directly binds at the IRF7 promoter and could control its transcription (Litvak et al., 2012). Another study suggested a negative regulatory role of FOXO1 in the cellular antiviral response by promoting the ubiquitination of IRF3 and subsequent IRF3 protein degradation (Lei et al., 2013). Both studies suggest the possible regulatory circuit that some FOXO proteins controlling IRF3 or IRF7 to prevent excessive innate immune response that could result pathological outcome. Differential expression of FOXO3, FOXO4 and FOXO6 as a result of the IRF7 overexpression (**Figure 3E** and **Supplementary Figure S2A**) may suggest possible conservation of the feedback circuits controlling the antiviral response associated with FOXO transcription factors in chickens.

The death receptor (DR) signaling pathway is another arm of the extrinsic apoptotic process (Orzalli and Kagan, 2017). There have been reports regarding the detrimental effects of IFNs in influenza virus infection in which excessive IFN levels lead to severe damage on the host (McNab et al., 2015). Influenza virus susceptible mouse strains were found to have a stronger and more sustained type I IFN signal than resistant strains and antagonizing the type I IFN signal in susceptible strains improved host survival and reduced inflammation (Davidson et al., 2014). It was suggested that the disease-promoting effects of IFN are possibly mediated by upregulation of apoptosis-inducing proteins such as TNF-related apoptosis-inducing ligand (TRAIL) and its receptor DR5 (TNFRSF10B) or Fas cell surface death receptor (FAS) which could lead to the tissue damage in somatic cells and immunosuppression on immune cells (Fujikura et al., 2013; Hogner et al., 2013; Davidson et al., 2014). In our study, *TRAIL* was not differentially expressed and downregulation of *DR5* and *FAS* (**Figures 2A,B**) were observed due to IRF7 overexpression, which suggests that the IRF7 mediated apoptosis may not utilize the DEATH receptor mediated mechanism.

Necroptosis is now recognized as an alternative to apoptosis as a mechanism of controlled cell death Necroptosis has a distinct regulation mechanism compared to unintentional cell death by necrosis and it is a better inducer of a strong proinflammatory response that is crucial to the host immune response when compared to the apoptosis (Mocarski et al., 2015; Orzalli and Kagan, 2017). A protective role of Receptor Interacting Serine/Threonine Kinase 3 (RIPK3) has been reported against viral infections including influenza A virus and RIPK3 is known as a key factor upon viral infection that determines whether the infected cells undergo apoptosis or necroptosis (Newton et al., 2014; Nogusa et al., 2016; Thapa et al., 2016; Daniels et al., 2017). In this study, *RIPK3* was also up-regulated by IRF7 overexpression and along with the increased cell death, this might suggest a novel functional role of chicken IRF7 as a potential regulator in the necroptosis pathway against AIV infection.

In addition, chicken IRF7 may regulate genes involved in the cell structural integrity or cellular assembly such as cell adhesion, ECM organization, and adherens junctions (**Figures 2E,F**, **3A,B**) which have a wide range of functions in the host response to viral infection. Actin cytoskeleton plays an important role in the entry of influenza virus into cells and proper assembly of viral particles (Sun and Whittaker, 2013; Kumakura et al., 2015). ECMs are also critical across many stages of the viral life cycle, including viral entry, transmission, and exit (Stavolone and Lionetti, 2017). Integrin mediated cell adhesion to ECM is essential for survival of many cell types (Meredith and Schwartz, 1997), and apoptotic cells undergo distinct morphological changes characterized by cell and nucleus shrinkage as well as disassembly into apoptotic bodies which are associated with structural proteins such as actins and laminins (Saraste and Pulkki, 2000; Suzanne and Steller, 2009). Actin initiates and mediates mammalian apoptosis via the intrinsic and extrinsic pathways and final degradation of actin filaments amplifies the apoptosis signaling cascade. Differential regulation of these structural proteins (mostly up-regulated by IRF7 overexpression, **Figure 3D**) may contribute to the increased apoptosis and viral production yet the precise mechanism remains to be further elucidated.

Furthermore, interaction analysis of IRF7 overexpression and H6N2 infection was performed to discover the genes that were differentially regulated during the infection due to IRF7 overexpression. The analysis not only reinforced our findings from the direct contrast analyses (**Figures 2**, **3**), which suggested the modulator role of IRF7 in a wide range of host responses to H6N2 infection, but also discovered additional genes and pathways that were possibly modulated by IRF7. For example, the JAK-STAT signaling pathway was not enriched in any of direct contrast analyses but was significantly enriched from the interaction DEGs. The JAK-STAT pathway is one of the key pathways in the type I IFN response and employs interferon-stimulated genes to inhibit virus infection by targeting the viral life cycle and regulate the host processes (Schneider et al., 2014; Majoros et al., 2017). In our study, Tyrosine kinase 2 (TYK2, one of the JAKs) and its negative regulators SOCS1, SOCS3 as well as anti-apoptosis genes BCL-XL and PIM1 were differentially regulated by IRF7 during the H6N2 infection.

In sum, overexpression of IRF7 resulted in higher viral replication as well as increased cell death in DF-1 cell lines. Although it is unclear in our *in vitro* model if increased viral replication due to IRF7 overexpression is beneficial to the host or to the virus, our results suggest potential modulator function of chicken IRF7 in the programmed cell death via TGF-beta-FOXO signaling axis in the host response. In addition, we revealed sets of candidate genes that IRF7 might regulate in the cellular structure organization, highlighting cell-cell adhesion processes that play an important role in both host response and viral life cycle. Chicken IRF7 was also involved in metabolic pathways that have known functions in antiviral response. An ongoing complete loss-of-function study of IRF7 by CRISPR-Cas9 will expand our knowledge on potential regulatory role of IRF7-dependent and -independent pathways on AIV infection in poultry. A genome-wide IRF7 binding study by ChIP-seq would also help to expand our knowledge by determining whether the DEGs were directly modulated by IRF7 binding or by the cascade of downstream type I IFN responses.

## Author Contributions

TK and HZ conceived and designed the experiments, contributed reagents, materials, analysis tools, analyzed the data, and wrote the paper. TK performed the experiments.

## Conflict of Interest Statement

The authors declare that the research was conducted in the absence of any commercial or financial relationships that could be construed as a potential conflict of interest.
