# Supplementary figures and images for: Overexpression of Chicken IRF7 Increased Viral Replication and Programmed Cell Death to the Avian Influenza Virus Infection Through TGF-Beta/FoxO Signaling Axis in DF-1
Source: Front Genet. 2018 Sep 25;9:415. doi: 10.3389/fgene.2018.00415 (PMC6190866; doi:10.3389/fgene.2018.00415)

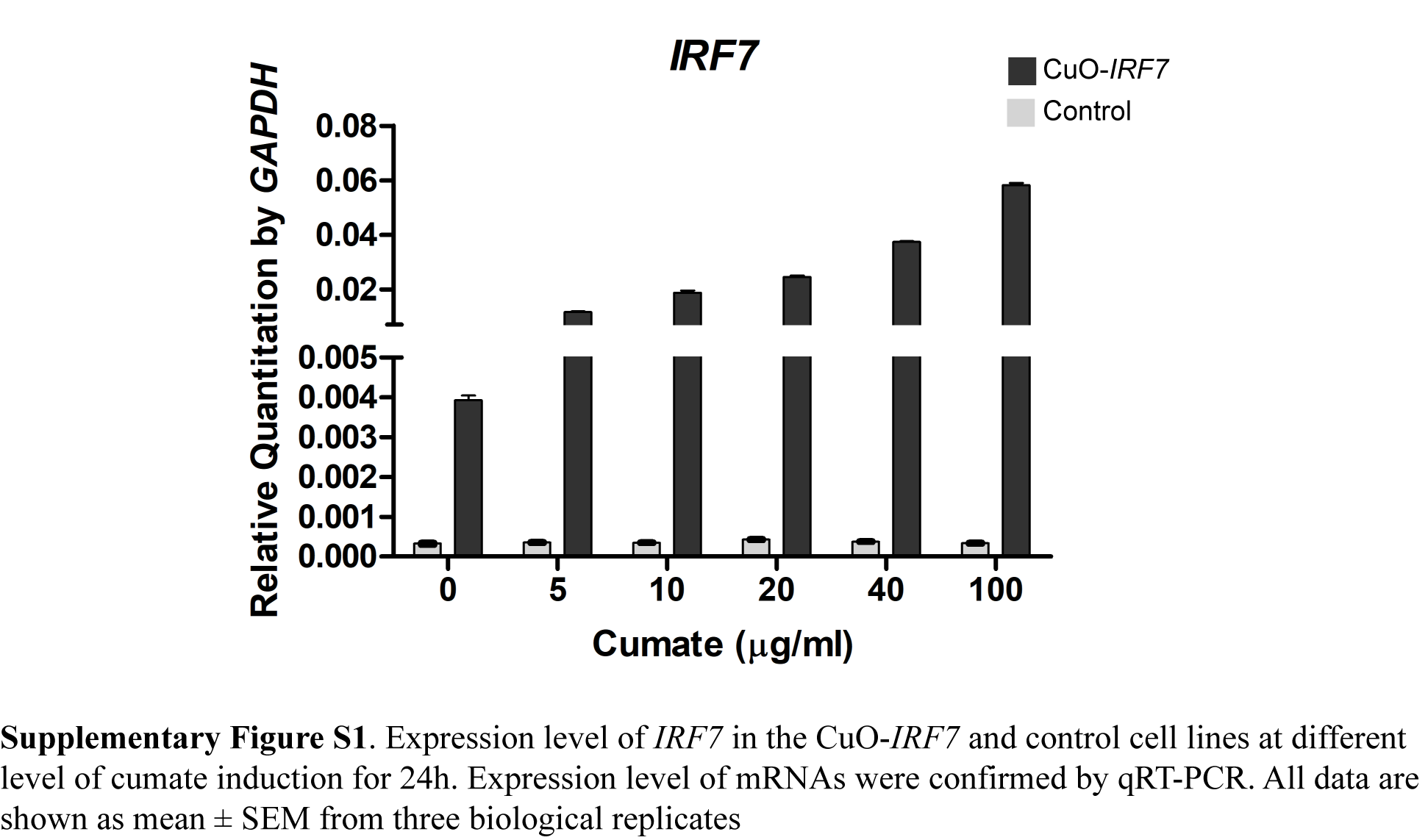

Supplement: Supplementary file 4 [file Image_1.TIF]

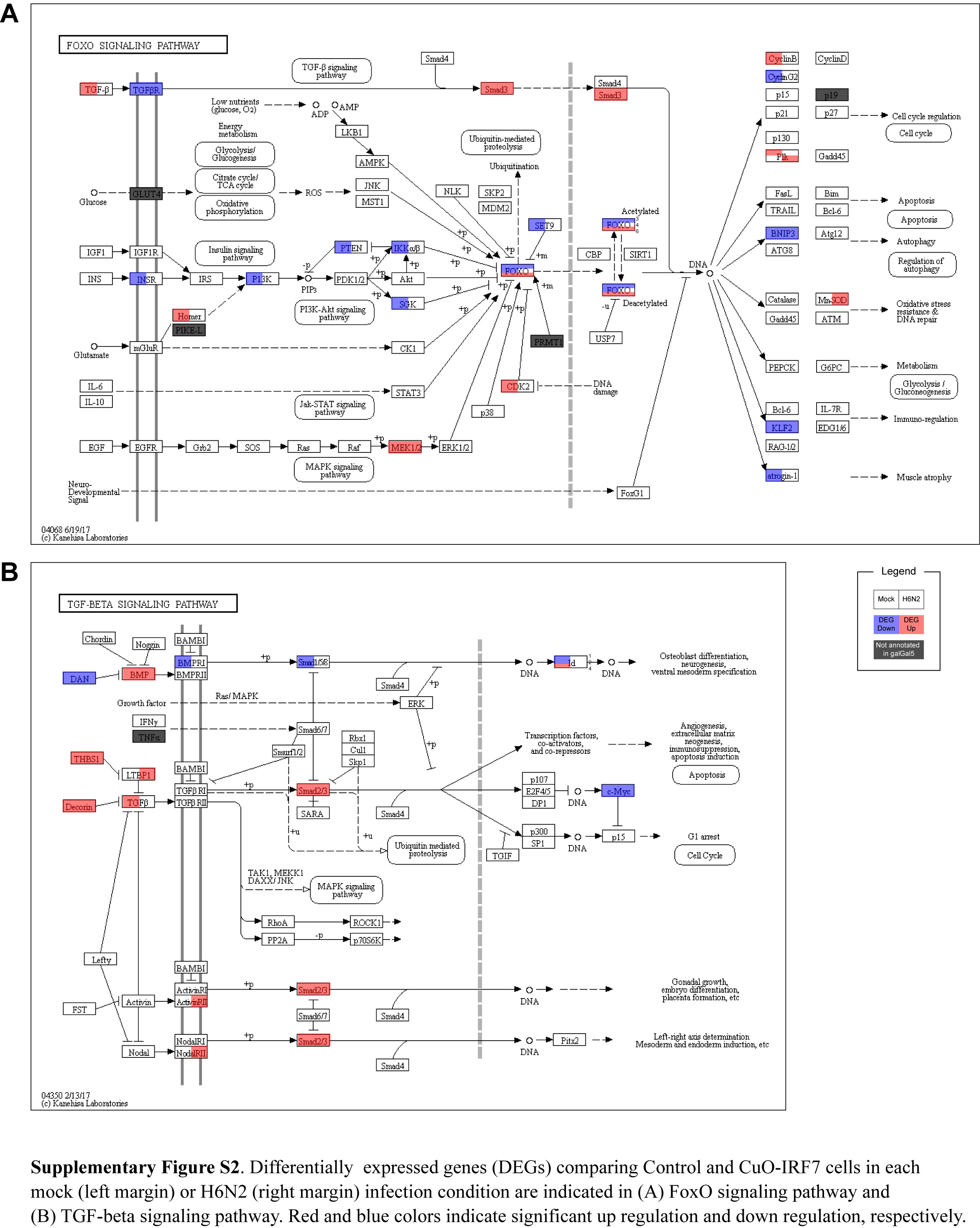

Supplement: Supplementary file 5 [file Image_2.TIF]

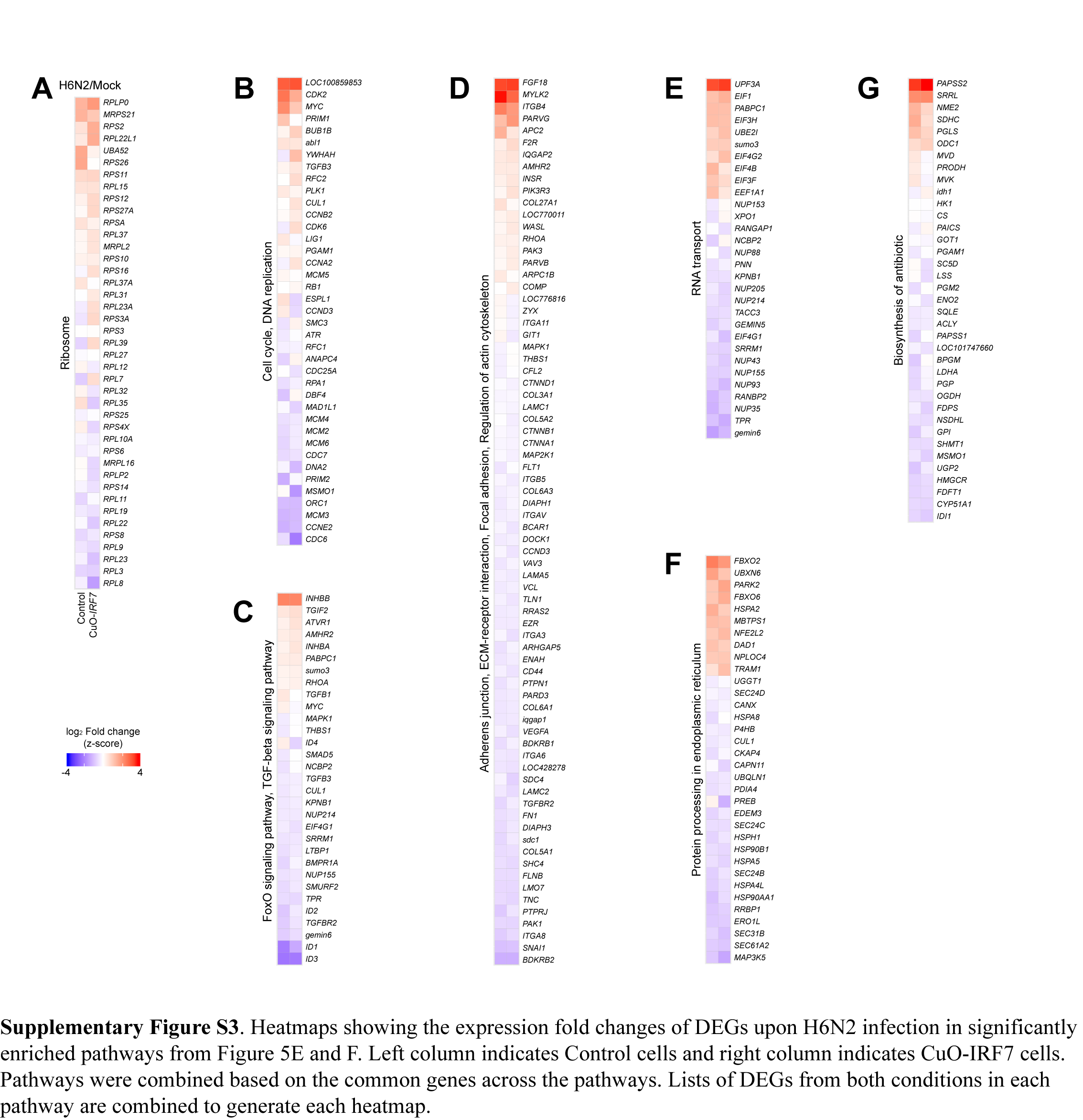

Supplement: Supplementary file 6 [file Image_3.TIF]
